# Prevalence and associated risk factors of gastritis among patients visiting Saint Paul Hospital Millennium Medical College, Addis Ababa, Ethiopia

**DOI:** 10.1371/journal.pone.0246619

**Published:** 2021-02-09

**Authors:** Zelalem Tadese Feyisa, Berhanu Teshome Woldeamanuel

**Affiliations:** 1 Department of Sociology, Salale University, Fitche, Oromia, Ethiopia; 2 Department of Statistics, Salale University, Fitche, Oromia, Ethiopia; Jamia Hamdard, INDIA

## Abstract

**Background:**

The health of individuals is not only the absence of disease checked medically, but also encompasses social and psychological aspects. Any departure from the state of physiological, psychological, or social well-being was affected by different factors. However, all contributory factors were not equally responsible for affecting disease. This study was undertaken as a search for the relative effects of sociocultural and individual behavioral factors contributing to acute and chronic gastritis patients visiting Saint Paul Hospital Millennium Medical College (SPHMMC).

**Methods:**

A cross-sectional study was carried out on 364 patients visited SPHMMC in the study. Primary data were collected through an interview schedule tool with an exit approach by validating questions pertaining to sociocultural and individual behavioral factors. The status of gastritis was measured as whether patients had *Helicobacter Pylori* infection, signs and symptoms indicated gastritis that occurred, and persisted for less than a month, greater than a month, or none of the signs and symptoms. Descriptive statistics, bivariate analysis, and multivariable ordinal logistic regression model were used to identify the predictors of gastritis severity. P-value ≤ 0.05 was declared as an indicator of statistically significant.

**Results:**

The prevalence of gastritis in the study area was 78.8%. Specifically, 48.9% and 29.9% had acute and chronic gastritis, respectively. The study found that low income and taking medicinal drugs was slightly significantly contributed to higher gastritis status; however, being younger age was slightly significantly contributed to lower gastritis status. Furthermore, the results indicated that eating spiced foods (Adjusted Odds Ratio (AOR) = 1.508; 95% CI: 1.046, 2.174), lack of physical exercise regularly (AOR = 1.780; 95% CI: 1.001, 3.168), stress (AOR = 2.168; 95% CI: 1.379, 3.4066), and substance use (AOR = 1.478; 95% CI: 1.093, 1.999) were significantly contributed to higher gastritis status.

**Conclusions:**

The findings suggested that women should take enough rest and sleep well, men refrain from involvement in any risky behaviors, young people and those who earn low income per month should equip with knowledge and understanding on how to practice good health behaviors, eating foods on time, avoiding eating spiced food frequently, doing physical exercise regularly, and taking medicinal drugs according to physician advice are recommended.

## Introduction

Gastritis is a disease which results from an inflammation of the gastric mucosa [1–3]. It is characterized by pain, swelling, and irritation of the mucosal membrane of the stomach [1]. Moreover, it manifested by a sign and symptom such as nausea, vomiting, dull pain, discomfort in the upper abdomen, feeling of fullness, and loss of appetite [1, 3, 4].

Gastritis is either an acute or a chronic depending on how long the signs and symptoms persist [1, 5, 6]. In particular, acute gastritis is an inflammation of the stomach lining that occurs suddenly and lasts shortly within one or two days and even less than a month [5, 7, 8]. Correspondingly, chronic gastritis is an inflammation of the gastric mucosa that occurs gradually and persists for more than a month and even for some years [9, 10].

Gastritis still remains a social and public health problem both in developed and developing countries [2, 9, 11]. It is an underlying cause affecting individuals’ socioeconomic status, health behaviors, and living standards such as lifestyles, living conditions, behaviors, and habits [12]. Globally, 50.8% of the populations in developing countries suffer from gastritis [13, 14]. With a lower figure, 34.7% of the population in developed countries had health problems due to gastritis [14]. Compared with developing countries, the prevalence rate of gastritis markedly decreased in developed countries. However, it has been remained as a major health problem [2, 15]. In general, gastritis was higher among men than women [16–18]. However, a study conducted in Brazil showed 67.8% of women and 32.2% of men suffered from chronic gastritis [19]. Furthermore, among patients who visited a public hospital in Brazil, 35.4% of adults above 40 years of age, and 24.7% of adolescents less than 40 years of age suffered from gastritis [14].

A systematic review of African countries indicated 38% of women and 18% of men suffered from gastritis. In Kenya, among patients who visited health care institutions, 73.3% of children and 54.8% of adults diagnosed clinically as they had gastritis. Similarly, in Uganda, 44.3% of young people less than the age of 12 years were suffering from gastritis. Furthermore, in Nigeria, 40.7% of children with an age range from 6–10 years had gastritis due to *H*. *Pylori* [3].

In Ethiopia, a systematic review carried out by Marcis et al. [14] indicated that 53% of individuals with the age range from 54–61 years had gastritis due to *H*. *Pylori* infection. Another study conducted in Hawassa University indicated that 67.8% of male students and 32.2% of female students suffered from gastritis [9]. Similarly, a study conducted in Jigjiga University showed male students are more suffering from gastritis than females [20]. Finally, as it was indicated by Demisew [9] in Ethiopia, gastritis was common among adolescents than old people.

An identification of particular associated risk factors of any social problem, including health is a critical point for applying the necessary measures [21]. Thus, differentiating the specific contributory factors of gastritis, either acute or chronic, helped to understand the relative effects of the factors [22]. In turn, comprehensively, such investigations helped in applying intervention measures on factors contributing gastritis to different severity levels.

Several previous studies found out factors such as sex, age, socioeconomic status, biological, environmental factors, and individual behaviors significantly contributed to gastritis [1, 3, 15, 23, 24]. Along these lines, to the best of our knowledge, none the studies in Ethiopia clearly identify the factors that contributed gastritis status which is either acute or chronic. Cognizant of such gaps and limited evidence in the study setting, this study aimed to assess the prevalence and relative effects of socio-cultural and individual behavioral factors of acute or chronic gastritis. Further, this study contributes, by adding value to the body of scientific knowledge, to identify the contributing factors of acute and chronic gastritis separate ways. While many studies have been done on the factors of gastritis in general, this study investigated the contributory factors of both acute and chronic gastritis separately. One of the main reasons for analyzing gastritis separately was its importance for interventions specifically and to identify the contributing factors and to publicize their prevalence.

## Theoretical framework of the study

This study used the Social Ecological Theory. As it was explained by Kuykendall [25], the theory was developed by a group of experts, namely, Kenneth McLeroy, Daniel Bibeau, Allan Steckler, and Karen Glanz in 1988. The theory draws its attention to the contributory factors of health problems by addressing disease or illness. It addressed the contributory factors of disease or illness from the psychosocial environmental elements. Moreover, the theory emphasizes to single out contributory factors with different scales or levels, aiming at suggesting appropriate measures for the intervention.

Initially, the theory underlines individual behavioral factors which encompass variables such as diet, substance use, age, past experiences, and knowledge or attitudes toward physical exercise. Second, socio-cultural factors such as gender, beliefs, traditions and type of foods, preferences and choices of meals, socioeconomic status, norms, and values within their way of life. Finally, institutional and community factors such as faith-based institutions influencing dietary choices, the nature of protection, access to fresh fruits and vegetables, recreational areas, and housing nature were included.

The theory presents individuals at a center point and it explains that health in general and disease in particular were influenced by individual behaviors, community, socio-cultural, and institutional factors. Therefore, this study employed the theory as a theoretical framework aimed to determine the social, cultural, and individual behavioral factors affecting gastritis in the study area. In addition, the study used the theory not only identifying the contributory factors, but also forwards the best possible measures that reduce or diminish the problems.

## Materials and methods

### Study design

Hospital based cross-sectional study was conducted from January, 2020 to March, 2020 at Saint Paul Hospital Millennium Medical College (SPHMMC). The hospital is located in Addis Ababa, the capital city of Ethiopia. Since the hospital was a referral hospital, different people from different localities and regions visited the hospital for health cures and treatments.

### Population and sample sizes

In this study, a total of 364 patients who visited the hospital for treatment from January 2020 to March 2020 were included. The estimated sample size was arrived at using the single population formula [26]. The simple random sampling technique was used to contact the patients at each clinic or ward after completing treatment.

### Data and variables

Primary data were collected using the World Health Organization (WHO) STEP wise approach to surveillance of non-communicable disease standard survey method [22]. The data were collected from patients using an interview schedule tool with an exit approach at each clinic or ward. The collection of primary data was done by trained and experienced enumerators. Different items of questions related to socio-demographic, socio-cultural and individual behavioral factors were employed to measure the associated factors of gastritis. Before the actual data collection, a pilot study of 50 non-sampled patients was employed in each clinic and ward to check for the reliability of the tools, and then the necessary adjustments were made.

Gastritis was measured by *Helicobacter Pylori* status (either positive or negative results) and actual clinical observation of signs and symptoms related to digestive system [1–3]. The status of *Helicobacter Pylori* was analyzed using both serology (antibody) test and stool (antigen) test. Clinically diagnosed gastritis was analyzed by signs and symptoms, such as nausea, vomiting, dull pain, heartburn, abdominal discomfort, and loss of appetite in line with the gastrointestinal tract. The studies conducted by Cecilia et al. [1] and Smith et al. [3] pointed out that nausea, vomiting, dull pain, heartburn, abdominal discomfort, and loss of appetite were the signs and symptoms of gastritis. This study measured patients’ gastritis status by underlining the length of time through which *Helicobacter Pylori* infection, the signs and symptoms were occurred and persisted. Thus, the outcome variable (Y) was gastritis status classified into ordinal responses as:

$$Y=\left\{ \begin{array}{l} 1\;if\;patients\;had\;no\;H.Pylori\;infection,\;signs\;and\;symptoms\;related\;to\;gastritis\;\\ \;2\;if\;patients\;had\;H.Pylori\;infection,\;the\;signs\;and\;symptoms\;that\;last\;with<a\;month\;\\ \;3\;if\;patients\;had\;H.Pylori\;infections,\;the\;signs\;and\;symptoms\;that\;last\;with>a\;month\; \end{array}\right.$$

Broadly, the independent variables were categorized into socio-demographic, sociocultural, and individual behavioral factors. The socio-demographic and sociocultural factors include age, sex, income level, stress, medicinal drug use, washing hands before eating food, eating uncooked type of food, and eating regularly the same food. Whereas, individual behavioral factors include the preferences to spiced foods, skipping and delaying meals, physical exercise, and substance use.

### Statistical analysis

Data were cleared and entered into EpiData version 3.1 and then exported to SPSS software version 20.0. The distribution of the study participants was presented using frequencies and percentages and the ordinal logistic regression model was used to identify the risk factors associated with gastritis status.

Pearson chi-square test was used in the bivariate analysis to test the association of each independent variable with the outcome variable. Then, all significant predictors with p-value < 0.25 in the bivariate analysis were included in the multivariate ordinal logistic regression analysis. A measure of how well the multivariable ordinal logit model fits the data adequately was checked using a likelihood ratio chi-square test at *p*-value <0.05. Furthermore, the model relies on the parallel regression assumption, which requires the effects of independent variables to be equal for different categories. In this study, the parallel regression assumption was assessed by using the likelihood ratio test. Finally, the modeling procedure was employed to test the existence of multicollinearity among independent variables using Variance Inflation Factor (VIF) with a cut point value that was set at 10. The odds ratio with 95% confidence intervals was reported for the significance of predictor variables to the outcome variable in multivariable ordinal logistic regression analysis.

### Ethics and consent to participate

The study was got an official permission letter from the Salale University Office of Vice President for Research and Community Services to do the investigation. Additionally, an official permission letter was obtained from the Office of Chief Executive Officer of SPHMMC for collecting data. Lastly, verbal consent from study participants was also obtained after explaining the purpose of the study and confidentiality was assured using the coding system; the interview schedule did not have any personal identifiers.

## Results

### Descriptive statistics of the sample characteristics

The result of descriptive analyses of the sample characteristics was presented in Table 1. Among the total study participants, 246 (67.6%) were females, while 118 (32.4) were males. Study participants’ age ranges from 18 to 56 years old. Nearly half of the study participants, 181 (49.7%) were between the age of 18–28 years old, 146 (40.1%) were between 40–50 years old, while only 21 (5.8%) and 16 (4.4%) were aged 29–39 and above 50 years old, respectively. The vast majority of the study participants 308 (84.6%) were lived in urban areas, while only 56 (15.4%) lived in rural areas. Moreover, the majority of the study participants 154 (42.3%) were attended college or higher education, 125 (34.3%) were attended primary school, while only 85 (23.4%) were completed high school.

**Table 1:** Descriptive analysis of variables with their categories, SPHMMC 2020 (n = 364).

Background characteristics	Categories	N	%
Sex	Female	246	67.6
	Male	118	32.4
Age	18–28	181	49.7
	29–39	21	5.8
	40–50	146	40.1
	>50	16	4.4
Place of residence	Urban	308	84.6
	Rural	56	15.4
Educational level	Below grade 9	125	34.3
	High school completed	85	23.4
	College and above	154	42.3
Monthly income level	<2000	94	25.8
	2001 to 4000	92	25.3
	4001 to 6000	85	23.4
	>6001	93	25.5
Marital status	Unmarried	161	44.2
	Married	147	40.4
	Divorced	36	9.9
	Widowed	20	5.5
Occupation	Unemployed	166	45.6
	Self-employed	79	21.7
	Governmental	85	23.4
	Non-governmental	34	9.3

Source: Authors Survey, 2020.

Related to monthly income the distribution was nearly consistent. About 94 (25.8%) of the study participants were earning less than 2000 ETB and 93 (25.5%) were earning 6001 ETB. Moreover, 92 (25.3%) were earned 2001 to 4000 ETB. Similarly, 85 (23.4%) were earned 4001 to 6000 ETB. The majority of the study participants 161 (44.2%) were unmarried, 147 (40.4%) of them were married, while only 36 (9.9%) and 20 (5.5%) were divorced and widowed, respectively. Nearly half, 166 (45.6%) of the study participants were unemployed, while nearly one-fourth, 85 (23.4%) were governmental employees, nearly one-fifth, 79 (21.7%) were self-employed and only 34 (9.3%) were non-governmental employees (Table 1).

### Prevalence of gastritis

As it was presented in Table 2, from a total study participant, 178 (48.9%) had acute gastritis and 109 (29.9%) had chronic gastritis. A stool antigen positive for *H*. *Pylori* was detected among 124 (34.1%) patients and a serology positive for *H*. *Pylori* was detected among 88 (24.2%) patients. In addition, 72 (20.6%) of the patients were diagnosed clinically based on the signs and symptoms as they had for gastritis (Table 2). Concerning gastritis status by demographic characteristics, more than half, 128 (52%) of female study participants had acute gastritis and nearly one-third, 77 (31.3%) had chronic gastritis, whereas 50 (42.4%) male study participants had acute gastritis and only 32 (27.1%) had chronic gastritis, respectively. Corresponding to age, 104 (57.5%) of the study participants between 18 and 28 years old had acute gastritis, while 58 (39.7%) of the study participants between 40 and 50 years old had gastritis. Similarly, 55 (37.7%) of the study participants aged 40 and 50 years old, and 43(23.7%) of the study participants aged 18–28 years old had chronic gastritis.

**Table 2:** Prevalence of gastritis and *Helicobacter Pylori* positivity, SPHMMC 2020 (n = 364).

Variables	Total Participants	Gastritis status		
		None	Acute	Chronic
	n(%)	n(%)	n(%)	n(%)
**Sex**				
Female	246(67.6)	41(16.7)	128(52)	77(31.3)
Male	118(32.4)	36(30.5)	50(42.4)	32(27.1)
**Age group (years)**				
18–28	181(49.7)	34(18.8)	104(57.5)	43(23.7)
29–39	21(5.8)	9(42.9)	10(47.6)	2(9.5)
40–50	146(40.1)	33(22.6)	58(39.7)	55(37.7)
>50	16(4.4)	1(6.2)	6(37.5)	9(56.3)
**Diagnosis**				
Stool antigen positive for *H*. *Pylori*	124(34.1)	-	86(69.4)	38(30.6)
Serology positive for *H*. *Pylori*	88(24.2)	-	57(64.8)	31(35.2)
Clinically diagnosed gastritis	75(20.6)	-	43(57.3)	32(42.7)
Without gastritis	77(21.1)	77	-	-

Source: Authors Survey, 2020.

### Bivariate analysis of associated factors and type of gastritis

A cross-tabulation of various characteristics of the study participants with gastritis status and a chi-square test of association were presented in Table 3. Among study participants, 128 (52%) and 77 (31.3%) of females had acute gastritis and chronic gastritis, respectively. Whereas, 50 (42.4%) and 32 (27.1%) of males had acute gastritis and chronic gastritis, respectively. Concerning place of residence, 152 (49.4%) and 26 (46.4%) of the study participants living in urban and rural areas, respectively, had acute gastritis while, 93 (30.2%) and 16 (28.6%) of the study participants residing in urban and rural areas, respectively, had chronic gastritis. On the other hand, as the educational level was concerned, 55 (44%) and 51 (60%) of the study participants who attended primary education and completed high school had acute gastritis, respectively. Whereas, 72 (46.8%) of the study participants who attended college and above had chronic gastritis. Nearly one-third, 40 (32%) and 24 (28.2%) of the study participants who attended primary education and completed high school, respectively, had chronic gastritis. In addition, 45 (29.2%) of the study participants who attended college or higher education had chronic gastritis (Table 3).

**Table 3:** Bivariate analysis of independent variables of gastritis status, SPHMMC 2020 (n = 364).

Variables	Categories	Status of gastritis						Pearson Chi-Square (p-value)
		None		Acute		Chronic		
		f	%	f	%	F	%	
Sex	Female	41	16.7	128	52	77	31.3	0.010
	Male	36	30.5	50	42.4	32	27.1	
Place of Residence	Urban	63	20.4	152	49.4	93	30.2	0.024
	Rural	14	25	26	46.4	16	28.6	
Educational Level	Below Grade 9	30	24	55	44	40	32	0.099
	High school Completed	10	11.8	51	60	24	28.2	
	College and above	37	24	72	46.8	45	29.2	
Monthly Income Level	<2000	27	28.7	40	42.6	27	28.7	0.020
	2001 to 4000	18	19.6	50	54.3	24	26.1	
	4001 to 6000	19	22.3	39	45.9	27	31.8	
	>6001	13	14	49	52.7	31	33.3	
Marital Status	Unmarried	47	29.2	84	52.2	30	18.6	0.049
	Married	14	9.5	71	48.3	62	42.2	
	Divorced	8	22.2	16	44.4	12	33.3	
	Widowed	8	40	7	35	5	25	
Occupation	Unemployed	34	20.5	82	49.4	50	30.1	0.327
	Self-employed	22	27.8	35	44.4	22	27.8	
	Governmental	10	12.7	47	59.5	22	27.8	
	Non-governmental	5	14.7	14	41.2	15	44.1	
The sign of Gastritis	Nausea and vomiting.	-	-	31	64.6	17	35.4	<0.001
	Dull pain	-	-	62	63.3	36	36.7	
	Heartburn	-	-	27	51.9	25	48.1	
	Abdominal discomfort.	-	-	25	65.8	13	34.2	
	Loss of appetite.	-	-	33	64.7	18	35.3	
Persistent of the symptoms	Less than 2 days	-	-	70	39.3	-	-	<0.001
	Less than 14 days	-	-	108	60.7	-	-	
	A month ago	-	-	-	-	26	23.9	
	Less than 6 month	-	-	-	-	42	38.5	
	Above six month	-	-	-	-	41	37.6	
Habitual practices	Eating Spicy foods	-	-	154	80.2	38	19.8	0.001
	Lack of physical exercise	-	-	170	84.2	32	15.8	
	Stress and anxiety	-	-	166	86.9	25	13.1	
	Regularly eat similar food	-	-	44	51.8	41	48.2	
Drugs for treatment	Yes	-	-	163	60.6	106	39.4	0.016
	No	-	-	15	83.3	3	16.7	
Not washing hands before eating	Yes	-	-	118	59.3	81	40.7	0.002
	No	-	-	60	68.2	28	31.8	
Skipped and delayed meals	Yes	-	-	144	64.3	80	35.7	0.001
	No	-	-	34	54	29	46	
Eating uncooked foods	Yes	-	-	26	57.8	19	42.2	0.292
	No	-	-	112	49.6	114	50.4	
Involvement in Substance Use	Yes	-	-	115	57.5	85	42.5	0.001
	No	-	-	63	72.4	24	27.6	

With respect to income level, 40 (42.6%) and 50 (54.3%) of the study participants who earned less than 2000 ETB per month and who earned 2001–4000 ETB per month, respectively, had acute gastritis. Further, 39 (45.9%) and 49 (52.7%) of the study participants who earned 4001 to 6000 ETB and greater than 6001 ETB per month, respectively, had acute gastritis. Moreover, only 27 (28.7%) and 24 (26.1%) of the study participants who earned monthly less than 2001ETB and 2001–4000 ETB per month, respectively, had chronic gastritis. Whereas, 27 (31.8%) and 31 (33.3%) of the study participants who earned 4001–6000 ETB and greater than 6001 ETB per month, respectively, had chronic gastritis. As marital status was concerned, 84 (52.2%) and 71 (48.3%) of the study participants who were unmarried and married, respectively, had acute gastritis. Whereas, 16 (44.4%) and 7 (35%) study participants who were divorced and widowed, respectively, had acute gastritis. On the other land, only 30 (18.6%) and 62 (42.2%) of the study participants who unmarried and married, respectively, had chronic gastritis. Correspondingly, 12 (33.3%) and 5 (25%) of the study participants who were divorced and widowed, respectively, had chronic gastritis (Table 3).

Regarding occupation, 82 (49.4%) and 35 (44.4%) of unemployed and self-employed study participants, respectively, had acute gastritis. Besides, 47 (59.5%) and 14 (41.2%) of the study participants who worked at governmental and non-governmental organizations, respectively, had acute gastritis. Moreover, 50 (30.1%) and 22 (27.8%) of unemployed and self-employed study participants, respectively, had chronic gastritis. Whereas, 22 (27.8%) and 15 (44.1%) of the study participants who worked in governmental and non-governmental organizations, respectively, had chronic gastritis. With reference to habitual practices, 154 (80.2%) study participants who ate spicy foods and 170 (84.2%) of those not exercised physical activity had acute gastritis. Moreover, 166 (86.9%) of the study participants who felt stressed and anxiety and 44 (51.8%) of those regularly eating similar food had acute gastritis. Apart from this, 38 (19.8%) of the study participants who ate spicy food and 32 (15.8%) of those not exercised physical activity had chronic gastritis. Furthermore, 25 (13.1%) of the study participants who felt stressed and anxiety and 41 (48.2%) of those who ate regularly similar food had chronic gastritis (Table 3).

Regarding drugs taken to a treatment, 163 (60.6%) of the study participants had acute gastritis because of taking drugs. Moreover, 106 (39.4%) of those who took the drugs had chronic gastritis. Furthermore, 118 (59.3%) of the study participants who did not wash hands before eating meals had acute gastritis and 81 (40.7%) study participants who had meals without washing hands had chronic gastritis. Concerning to meals, 144 (64.3%) of the study participants who ate meal skipping and with delaying had acute gastritis. Apart from this, 80 (35.7%) of those who ate meal skipping and delayed had chronic gastritis. Furthermore, 26 (57.8%) of the study participants who ate uncooked foods had acute gastritis and 19 (42.2%) had chronic gastritis. Relating to substance use, 115 (57.5%) and 85 (42.5%) study participants who used the substance had acute gastritis and chronic gastritis, respectively (Table 3).

Regarding the symptoms of gastritis, 31 (64.6%) of the study participants who had nausea or vomiting symptoms and 62 (63.3%) the study participants with a dull pain symptom had acute gastritis. The results also revealed that 27 (51.9%) of the study participants had heartburn symptoms and 25 (65.8%) of those with an abdominal discomfort symptom had acute gastritis. Furthermore, 33 (64.7%) of the study participants with the loss of appetite symptoms had acute gastritis. Correspondingly, 17 (35.4%) of the study participants with nausea or vomiting symptoms and 36 (36.7%) with dull pain symptoms had chronic gastritis. It also revealed that 25 (48.1%) of the study participants with heartburn symptoms and 13 (34.2%) of those with an abdominal discomfort symptom had chronic gastritis. On the other hand, 18 (35.3%) of the study participants with a symptom of loss of appetite had chronic gastritis (Table 3).

### Goodness fit of the model

The VIF statistics for multicollinearity test were less than 10 for all variables, indicating that there is no collinearity between the predictor variables. Furthermore, the assumption of parallelism was checked using the likelihood ratio test (18.133, p-value = 0.201), and it implied that the largest surfaces are parallel, indicating that the model fits well. Likelihood ratio test (chi-square = 55.009, p-value< 0.001) indicated the model good fits. The -2 log likelihood value of the final model (627.872, p-value <0.001) is smaller than that of the initial model (646.005), indicating that the model fits the data well. Furthermore, the ratio of value to the degree of freedom of Pearson statistics is close to 1, revealing that the model fits well (Table 4).

**Table 4:** Goodness of fit for ordinal logistic regression analysis, SPHMMC, 2020 (n = 364).

Index	Value
Test of Parallel Lines	0.201
Likelihood Ratio Test	χ2 = 14.074, p <0.001
-2 Log Likelihood	627.872, p-value <0.001
Pearson Chi-square	χ^2^ = 626.401, v/df = 1.111

### Factors associated with gastritis

The multivariable ordinal logistic regression analysis was presented in Table 5. The result indicated that being female was 0.655 (AOR = 0.655; 95% CI: 0.503, 0.852) times less likely to be at higher gastritis status than males. Moreover, study participants with higher age were slightly significant to be at higher gastritis status than those of lower age. Apart from this, study participants of low income were slightly significant to be at higher gastritis status than those of higher income level. Likewise, study participants eating spicy foods was 1.508 (AOR = 1.508; 95% CI: 1.046, 2.174) times more likely to be at higher gastritis status than those eating regularly similar foods. Furthermore, study participants who did not involve in physical activity were 1.780 (AOR = 1.780; 95% CI: 1.001, 3.168) times more likely to be at higher gastritis status than those exercising with slight significance.

**Table 5:** Multivariate ordinal regression analysis for type of gastritis, SPHMMC2020(n = 364).

Variables	B	S.E	Sig.	AOR	95% CI for AOR	
					Lower	Upper
**Sex**						
Female	-.423	.1342	.002*	.655	.503	.852
Male	0^a^	.	.	1	.	.
**Age** (Years)	-.010	.0056	.074**	.990	.979	1.001
**Residence**						
Urban	-.174	.1678	.301	.841	.605	1.168
Rural	0^a^	.	.	1	.	.
**Monthly income level (ETB)**^**b**^						
< 2000	.348	.1822	.056**	1.416	.991	2.023
2001–4000	.211	.1715	.218	1.235	.883	1.729
4001–6000	.209	.1736	.229	1.232	.877	1.732
>6001	0^a^	.	.	1	.	.
**Nature of lifestyles**						
Eating spicy foods	.411	.1866	.028*	1.508	1.046	2.174
Lack of physical exercise	.576	.2943	.050*	1.780	1.001	3.168
Stress and anxiety	.774	.2306	.001*	2.168	1.379	3.406
Eating regularly similar foods	0^a^	.	.	1	.	.
**Taking medicinal drugs (yes)**	.400	.2356	.090**	1.492	.940	2.368
	0^a^	.	.	1	.	.
**Not washing hands before eating (yes)**	-.026	.1561	.866	.974	.717	1.323
	0^a^	.	.	1	.	.
**Skipped and delayed meals (yes)**	-.303	.1503	.044*	.738	.550	.991
	0^a^	.	.	1	.	.
**Eating uncooked foods (yes)**	.017	.1350	.901	1.017	.780	1.325
	0^a^	.	.	1	.	.
**Substance use (yes)**	.391	.1539	.011*	1.478	1.093	1.999
	0^a^	.	.	1	.	.

*significant at p<0.05.

**slightly significant at p<0.05.

ETB: Ethiopian Birr.

Similarly, study participants who had stress and anxiety were 2.168 (AOR = 2.168; 95% CI: 1.379, 3.406) times more likely being at a higher gastritis status than those without stress. Moreover, study participants taking medicinal drugs for treatment were slightly significant to be exposed to higher gastritis status than those not taking medicinal drugs. Furthermore, study participants who did not skip and delay their meals were 0.738 (AOR = 0.738; 95% CI: 0.550, 0.991) times less likely to be at higher gastritis status than those skipped and delayed their meals. Finally, study participants who involved in substance use were 1.478 (AOR = 1.478; 95% CI: 1.093, 1.999) times more likely to be at higher gastritis status than those not involved.

## Discussion

The prevalence of gastritis among the study participants was 78.8%, i.e., 48.9% and 29.9% had acute and chronic gastritis, respectively. The results indicated a higher number of women were suffering with gastritis than men. This result agrees with the studies conducted by Smith et al. [3], Jannathul et al. [12], and Agbor et al. [24]. Conversely, the result disagrees with the findings of [16–18]. This high prevalence of gastritis among females in this study might be due to females are more likely to visit health facilities than males seeking care gastritis and other health problems. As long as the age was concerned, the results showed that (55.5%) of the study participants less or equal to 39 years old suffered from gastritis, while 44.5% who were aged above 39 years old suffered from gastritis. This result disagrees with the findings of [14] which reported a higher (35.4%) of gastritis among 39 or higher years old individuals, while only 24.7% of those less than 40 years old were suffering from gastritis. Likewise, the result was in contrast to the study carried out in Switzerland [16]. Overall, the inconsistence of the results with previous findings might be as a result of the methodologies, survey instruments, sampling strategies employed, or study population.

The ordinal logistic regression results indicated being female was associated with decreased odds of gastritis severity compared with males. Similarly, prior studies [2, 13] and a meta-analysis done forty different studies [16] indicated that men were suffering from gastritis more than women. Furthermore, a study carried out in China [18] indicated the number of men affected by gastritis than women. However, the systematic review analysis in Africa [3] reported more number of women (38%) than men (18%) were suffered with gastritis. The results indicated, a higher number of women were suffering from acute gastritis than chronic gastritis. In other words, more number of men were suffered from chronic gastritis than acute gastritis. The studies conducted by Maeda et al. [27], and Janzen & Kelly [28] indicated that domestic works were mainly carried out by women. According to Barkan [29], gender was affecting the health status and behavior of individuals. Along this line, women generally engage in positive health behaviors, while men are more likely to engage in risky behaviors. The works are characterized by more time-spending activities, full of distress and stress, doing similar tasks repeatedly, and viewed as an inherently negative activity. In a male-dominated society like Ethiopia, women are more involved in domestic work, including serving and caring for the whole family. Owing to differences in social roles, women lived in stressful setups, had less time for taking rest, wakening up early from bed, and later going to bed for sleeping, faced boredoms physically with instable emotional and psychological arrangements. For this reason, women were visited nearby health care institutions for their illness or disease including gastritis than men. Another possible explanation might be Ethiopia men visit health facilities after severely affected and they are highly consuming substance and illicit drugs. As a result of such differences, women were less likely to develop chronic gastritis than men; due to experiencing more masculine characteristics; men were more suffered with chronic gastritis than acute.

It was found that being older was associated with slightly decreasing the odds of the gastritis status compared to younger. In other words, the study participants who were younger experienced more acute gastritis than chronic gastritis. Several studies found out that younger individuals were suffered from gastritis [1, 6, 20, 30]. Conversely, in China [31] the likelihood of getting gastritis among young and adults were common; nevertheless, in the young it was asymptomatic. In other words, gastritis was more prevalent among older than younger people. All aforementioned studies generally explained about the prevalence of gastritis, although they did not illustrate its severity level in a separate way. Like gender, age affects the status of health and disease patterns due to social factors [32]. As explained by Maeda et al. [27], a person’s interpretation of the social situation can be changed through continued experience and interaction. Along these lines, older people had the experiences; they obtained different knowledge that guided them on how to practice good health behaviors. For such practices, the likelihood of older people suffering from acute gastritis was less compared with younger adults.

Further, it was found that earning low income was associated with slightly significantly increased odds of being in chronic gastritis compared with earning high income. Study participants who earned a higher income per month were less likely to suffer from chronic gastritis compared with those earned less. Previous studies [3, 14, 23, 24] explained the general association of income with gastritis, although they did not clarify the association of income level with gastritis in specific ways. This might be people with less income level were less likely to visit health care institutions [33]. Moreover, they had less ability to pay for health services due to they had other life expenses. The social status of individuals within a social structure was determined by their income level. Owing to such circumstances, the probability of individuals who had low income to be exposed to risky health behaviors that contribute gastritis was high.

Eating spiced foods were another important variable, which increased the odds of the severity of gastritis. This study identified individuals who ate spiced foods were more likely suffered chronic gastritis than acute gastritis. The odds of chronic gastritis were estimated to be about two times higher among the study participants who were eating spiced foods than those who were eating regularly the same foods. The findings of the studies [4, 12, 14, 18] indicated that eating spiced foods resulted in gastritis, even though they did not identify the type of gastritis which is either acute or chronic. This might be due to the spiced food has a flavor or fragrance than other foods; it was commonly preferred to eat. In addition to such special properties, the spiced food has the potential to inflame and burn a gastric mucosa. Furthermore, such type of foods had inadequate nutrition and without balanced diets. The increasing number of individuals eating soft foods enriched with different flavors, aromatic and spicy, was also counted as a major factor.

The results showed that lack of physical exercise increased the odds of being in chronic gastritis by a factor of 78%. The study conducted in Sri Lanka [2] indicated as gastritis paralleled with lack of physical exercise. This might be physical exercise may refrain individuals from involvement in risky behaviors; in turn, help to have healthy lifestyles without risk behaviors or conditions. Doing physical exercises can help one to maintain the social well-being with healthy spirit, physical and mental well-being, too. Thus, if individuals are health physically and mentally, including spiritually, the probability of exposure to illness is less. Further, as described by Barkan [29], practicing physical exercise regularly was one of the elements of health-protective behavior.

It was also found that the odds of gastritis severity were 2.168 times higher among study participants who had stresses. Previous studies [1, 9, 12, 34] indicated that stress was the major risk factor for gastritis, although they did not clarify the type of gastritis which is either acute or chronic resulting from the stresses. Hereafter, the current study investigated that stress had a potential to cause chronic gastritis than acute gastritis. Among the social circumstances, negative life events, poverty, overcrowding, and traumas on mental and physical health were mentioned as major. Stresses which emanate from such circumstances have impaired physical and mental health and in particular can lead to chronic diseases like gastritis and other problems [35].

The medicinal drugs taken for treatment were another variable that slightly significant in contributing to chronic gastritis. Earlier studies [2, 9, 10, 36] stated that the medicinal drugs taken for treatment were significantly associated with gastritis; however, they did not elucidate which types of gastritis were resulted from the drugs. As a consequence, the current study identified the medicinal drugs which taken for treatment contributed to chronic gastritis than acute gastritis. The medicinal drugs which taken by individuals were nonsteroidal anti-inflammatory drugs (NSAID). Drugs such as aspirin and ibuprofen were mentioned as the major one. Such types of drugs have a potential to irritate the gastric mucosa and they have taken longer time for the treatment purpose, this may contribute to chronic gastritis.

Further, the odds of gastritis severity were more likely among individuals who had skipped and delayed meals. Individuals who ate their meals by skipping or delaying were more suffered from acute gastritis than chronic gastritis. Similar studies [12, 20] stated as gastritis developed from having meals by skipping, missing, and delaying ways from the usual one. As it was enlightened on the study [29], a medical sociologist, Salazar (2013) explained that having a proper time for each meal was essential for maintaining health and helping ill people regain their health. If not, risky behaviors, negative life events, and harmful behaviors related to eating meals could harm health and result in developing the disease.

Lastly, it was found that involvement in substance use is the practicing of smoking a cigarette, chewing khat, or using alcoholic beverages increases the odds of being in higher gastritis status. The studies [4, 7, 12, 16, 20, 24] stated that substance use aggravated the irritation or inflammation of the gastric mucosal. However, the type of gastritis that developed by involvement in substance use was not identified by the previously cited studies. As a result, individuals involved in risky health behaviors such as frequent cigarette use, alcohol abuse, and chewing, khat, individuals developed health problems. The CDC in 2011 [37] illustrated risky behaviors such as smoking, poor nutrition, physical inactivity, and using alcohol results in chronic diseases.

## Study strengths and limitations of the study

The current study was aimed to assess the prevalence and relative effects of sociocultural and individual behavioral factors of acute or chronic gastritis. The main strength of this study is that the reliability of the gastritis survey that was used and employed to identify the associated factors was derived from the World Health Organization (WHO) STEP wise approach to surveillance for non-communicable disease standards. Meanwhile, another strength is an investigation of the relative effects of sociocultural and individual behavioral factors on either acute or chronic gastritis.

One of the limitations is the potential sampling bias due to the use of simple random sampling, which might not be representative of the entire patients visiting the hospital. However, an attempt was made to reduce the sampling bias by distributing the survey to each ward or clinical setting with the consideration of patients coming from urban and rural areas and as well as the survey was distributed for lengthy periods. Meanwhile, another limitation is that the accuracy of the data is limited due to the use of a self-report survey, although it is considered a valid method of assessing the associated factors of either acute or chronic gastritis. Thus, the current results should be interpreted with caution.

## Conclusions

The results of the current study showed that being male, lower age, and skipping and delaying meals were contributed to acute gastritis. Whereas, low income per month, eating spiced of foods, and lack of physical exercise regularly were contributed to chronic gastritis. Furthermore, social stresses, taking medicinal drugs to treatment, and involvement in substance use were contributed to chronic gastritis. Women should take enough rest and sleeping and men should refrain from involvement in any risky behaviors resulting in stresses. Individuals, especially, the young and those who were earned low income should equip with knowledge and understanding on how to practice good health behaviors, should eat foods at proper times for each meal (breakfast, lunch, or dinner) and avoid eating spiced food frequently, should do physical exercises regularly, and should take their medicinal drugs after each meal or with other drugs that reduce the irritation of gastric mucosa. Another implication of this study is that, health information on the contributory factors of each type of gastritis is needed to create awareness and knowledge for individuals Finally, the authors recommended for future studies to incorporate more other contributing factors, more number of patients, more number of patients from rural areas, and both men and women by addressing their experiences and views of visiting health care institutions for illness or disease.

## Supporting information

S1 Data (RAR)

## Abbreviations

SPHMMC: Saint Paul Hospital Millennium Medical College
